# About Sugar Addiction

**DOI:** 10.1002/brb3.70338

**Published:** 2025-07-14

**Authors:** Di Qin, Jiayu Qi, Fuqiang Shi, Zhihua Guo, Hongwu Li

**Affiliations:** ^1^ Department of Geriatrics China‐Japan Union Hospital of Jilin University Changchun China; ^2^ School of Chemical Engineering Changchun University of Technology Changchun China

**Keywords:** high‐sugar food, pharmacological interventions, psychological interventions, sugar addiction, treatment of addiction

## Abstract

**Purpose:**

Sugar addiction, characterized by excessive cravings for high‐sugar foods, poses a significant health challenge in modern society. The parallels between sugar and drug addiction have attracted attention, prompting extensive research on the neurological mechanisms involved.

**Method:**

This review summarizes recent research on sugar addiction and its impact on health.

**Finding:**

High‐sugar consumption activates the brain's reward circuits—a positive reinforcement mechanism—including the dopamine and endorphin systems, which are associated with satisfaction and pleasure. Chronic exposure to high‐sugar foods may alter these systems, leading to heightened cravings and a dependence on sugar. Appetite is regulated by complex neural pathways, including those in the prefrontal cortex, amygdala, and hypothalamus. Therefore, dysfunction of these areas may lead to excessive sugar intake and impulsivity. Sugar addiction also negatively affects physical health, with high‐sugar intake strongly associated with obesity, diabetes, cardiovascular diseases, and other health problems. Sugar addiction can lead to weight gain and metabolic disorders, which in turn increase the risk of developing these diseases. Pharmacological and psychological interventions are the primary treatment for sugar addiction. Certain medications can reduce sugar cravings and dependence; however, their long‐term efficacy and side effects require further investigation. Cognitive behavioral therapy can also help individuals change their attitudes and behaviors toward sugar, thereby enhancing their self‐control.

**Conclusion:**

In summary, sugar addiction is an increasingly prevalent problem involving multiple aspects of the nervous system, behavior, and health. Further research on sugar addiction will enhance understanding, inform preventive and treatment measures, and elucidate the mechanisms underlying sugar addiction.

## Graphical abstract

This review synthesizes recent advances in understanding sugar addiction with a focus on its neurobiological mechanisms, health implications, and potential therapeutic interventions.

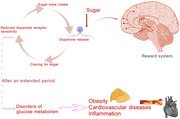

## 1 Introduction

### 1.1 Definition of Sugar Addiction

The new psychiatric classification system, the International Classification of Diseases 11th Revision (ICD‐11), has introduced the concept of behavioral addiction. However, it has also become clear that other behaviors, such as excessive consumption of palatable foods, can also lead to addiction. Food addiction is defined as a behavioral addiction characterized by the compulsive consumption of highly palatable foods, such as chocolate and sugary drinks, which is accompanied by the activation of the brain's reward system. The term “sugar addiction” reflects a notable physical or psychological dependence on high‐sugar levels or an increasing craving for sugary foods (Onaolapo et al. 2020).

Excessive sugar consumption is associated with complex behavioral disorders involving many genetic, biochemical, and environmental connections, similar to the chronic abuse of substances. Notable similarities exist between behavioral traits linked to long‐term substance dependence and excessive sugar intake, including compulsive overindulgence, cravings, loss of control, and withdrawal symptoms. Numerous attempts have been made to simulate the various facets of sugar overconsumption in rodents. For example, frequent sugar overindulgence can result in a condition in which the administration of opioid antagonists alleviates behavioral and neurochemical symptoms. Given that sugar intake induces cross‐sensitization to amphetamines, rats that consume sucrose exhibit greater sensitivity to psychostimulants than the control animals. Similarly, female mice fed sucrose developed locomotor sensitization to cocaine more rapidly than control animals fed only cocaine. In addition, operational definitions and behavioral evidence support each of the four components of addiction: overeating, withdrawal, seeking, and cross‐sensitization. Given that these actions are linked to the same neurochemical alterations in the brain as those observed in addictive substances, the concept of sugar addiction holds considerable validity.

## 2 Causes of Sugar Addiction

The peripheral organs of taste include taste buds located in the tongue, palate, and epiglottic epithelium. These cellular clusters resemble garlic bulbs and are situated in the fungiform, lobular, and circular papillae, which are found in the anterior, lateral, and posterior tongue areas, respectively. Taste receptor cells (TRCs) within taste buds possess G protein‐coupled receptors (GPCRs) that are sensitive to umami, bitter, and sweet flavors. TRCs respond to chemical stimuli dissolved in saliva (Depoortere 2014). Upon activation by sweet ligands, GPCRs activate chemosensory signaling pathways (Chaudhari and Roper 2010), which subsequently activate purinergic receptors on afferent fibers within the taste bud, thereby transmitting signals to the taste perception region of the gustatory cortex in the brain (Depoortere 2014). Moreover, chemical sensors in the gut and post‐absorptive sites, such as the pancreas, liver, and brain, can detect sugars. The vagus nerve conveys taste information from the gut to the small cell part of the ventral posterior medial nucleus of the thalamus, which subsequently relays this information via the ventral pathway to the amygdala and lateral area of the hypothalamus. In rodents, this information reaches the parabrachial nucleus (Fernstrom et al. 2012; Scott and Small 2009).

In rats and humans, intragastric or duodenal sugar consumption is linked to post‐enhancement of conditioned flavor preferences, as demonstrated in studies investigating potential sugar addiction. Notably, this sugar‐conditioned flavor preference can persist for days after its establishment, indicating that sugar conditioning enhances long‐term reward mechanisms (Onaolapo et al. 2020).

Sugar is energy‐rich and can rapidly elevate blood sugar levels, causing the release of insulin, which restores blood sugar to normal levels. However, excessive blood sugar can trigger fluctuations that may result in hypoglycemia, which in turn causes sugar cravings. Intermittent sucrose administration improves cholinergic, opioid, and dopaminergic neurotransmission in the mesocortical limbic system at the neurochemical level (Avena, Bocarsly, et al. 2008). Similar to substance misuse, prolonged sucrose consumption changes the ability of the nucleus accumbens (NAc) to release dopamine, and binge sugar consumption results in increased dopamine release in the NAc (Rada et al. 2005). Chronic sucrose consumption also affects nicotinic acetylcholine receptor (nAChR) expression in the NAc, and the effects of nAChR compounds on sucrose intake vary depending on the duration of exposure (Shariff et al. 2016).

Food cravings are also associated with imbalances in hormones, including the stomach hormone (ghrelin), hormone leptin (leptin), and neurotransmitter 5‐hydroxytryptophan (serotonin). These cravings for particular foods and substances are believed to indicate addiction patterns (Onaolapo et al. 2020). Peptide hormones that regulate sugar metabolism and consumption include growth hormone‐releasing peptides and leptin. Leptin is secreted by adipocytes, while hunger hormones are produced by gastrointestinal endocrine cells (Zhang et al. 2005). Both hunger hormones and leptin can cross the blood–brain barrier, influencing hypothalamic melanocortin system functions within the arcuate nucleus (ARC). Leptin activates pro‐opiomelanocortin (POMC)/cocaine‐ and amphetamine‐regulated transcript (CART) neurons, leading to stress‐related behaviors such as decreased food intake, weight loss, decreased α‐melanocyte‐stimulating hormone (α‐MSH) release, and decreased neuropeptide Y (NPY) release. In contrast, growth hormone‐releasing peptides activate NPY/agouti‐related peptide (AgRP) neurons, resulting in an increase in food intake, body weight, and AgRP release accompanied by a decrease in α‐MSH release. Leptin promotes weight loss by inhibiting food intake, whereas gastric starvation hormones act as appetite‐stimulating signals. High‐sugar diets, rich in carbohydrates, increase leptin levels and decrease circulating ghrelin levels. Growth hormone‐releasing peptides, along with leptin, insulin, and appetite‐regulating hormones, play key roles in the maintenance of energy homeostasis and the development and maintenance of behaviors (Bumb et al. 2019).

Sugar intake requires mutual regulation of the nervous and endocrine systems to maintain homeostasis in the body. The addictive properties of sugar are enhanced by its interaction with the brain's reward system. It is widely believed that drugs exert their effects on neural pathways that have evolved in response to natural rewards such as food, drink, and sexual activity. Key brain regions involved in these reward pathways include the middle forebrain bundle, nucleus ambiguus, ventral tegmental area, ventral medial and lateral hypothalamic nuclei, and the amygdala (Avena, Rada, et al. 2008). These areas play critical roles in processing and responding to natural rewards.

The brain reward system, also known as the mesocortical limbic circuit, operates based on brain structures and neural pathways linked to rewards, motivation, and the desire for pleasure (Yamaguchi et al. 2011). This reward system comprises interrelated structures such as the NAc, prefrontal cortex, hippocampus, amygdala, and ventral tegmental area (Arias‐Carrión et al. 2010). Excessive intake of glucose or fructose in humans leads to changes in the activity of brain regions associated with eating habits and rewards. Although glucose affects a wide range of brain functions, the effects of fructose are limited to neuronal activity (van Opstal et al. 2019). High‐sugar diets lead to excessively elevated glucose levels in the brain, which may cause changes in eating behavior.

Neurotransmitters in the brain such as dopamine, glutamate, and norepinephrine play important roles in appetite regulation. These neurotransmitters regulate appetite, satiety, and the reward mechanisms that govern food intake. For example, the dopamine system is associated with appetite and reward, and its activity affects food craving and intake in several ways. Dopamine is essential for controlling the mutually reinforcing effects of food and drugs. Long‐term substance abuse or excessive food consumption can lead to permanent alterations in the sensitivity and function of several neurotransmitter systems, including dopamine (Collins et al. 2015). Moreover, the hypothalamus and thalamus are important brain structures involved in appetite regulation. The hypothalamus promotes appetite by releasing signaling molecules such as NPY and orexin, whereas the thalamus suppresses appetite by releasing signals such as prostaglandin E2.

Other factors that contribute to sugar addiction include environmental and psychological factors that also affect appetite regulation. For example, sights, smells, and social factors can trigger appetite, whereas emotional states such as stress and anxiety may alter eating behaviors. High‐sugar foods are usually consumed during periods of negative mood, and in today's fast‐paced environment, individuals are more susceptible to stress, leading to increased consumption of high‐sugar foods. The activity of the nervous system is influenced by changes in the external environment and emotional state of the individual, affecting the intake of high‐sugar foods.

### 2.1 Dopamine

The dopamine system is essential to control the effects of meals and medications. The function and sensitivity of several neurotransmitter systems, including dopamine, can be irreversibly altered by long‐term substance abuse or excessive food consumption (Collins et al. 2015). Dopamine is a key neurotransmitter in the brain that controls pleasure and reward. Upon consumption, sugar is rapidly absorbed and converted to glucose, leading to the release of dopamine. In sham‐fed mice, sucrose consumption resulted in a linear increase in dopamine release within the nucleus ambiguus. In these animals, sucrose solution was introduced into the mouth and subsequently expelled through a cannula (Avena, Bocarsly, et al. 2008; Avena, Rada, et al. 2008). Hajnal and Norgren (2001) reported that dopamine release in the nucleus ambiguus increases with spontaneous licking of sugar. Notably, interfering with dopamine receptors in the nucleus ambiguus did not decrease sucrose uptake, and local infusion of nomifene resulted in increased dopamine levels and sucrose uptake.

Dopamine release activates the reward circuits in the brain, leading to feelings of pleasure and satisfaction. This pleasure reinforces memory and cravings for sugar, prompting continued seeking and consumption of sugary food. This creates a vicious cycle: ingesting sugar releases dopamine, which in turn results in feelings of pleasure, reinforces sugar craving, and subsequently leads to re‐ingestion of sugar and further dopamine release. Low dopamine levels in the brain may lead to withdrawal symptoms, which may induce continued sugar consumption, potentially leading to addiction. However, compared to other addictive substances, sugar leads to milder withdrawal symptoms. Specifically, individuals do not exhibit overt signs of sugar “addiction,” nor do they experience severe physical or life‐threatening withdrawal symptoms. However, this does not imply that sugar withdrawal does not occur in the brain. Notably, symptoms associated with attention‐deficit hyperactivity disorder (ADHD), including hyperactivity, inattention, distractibility, and poor performance, can be attributed to dopamine deficiency in the brain, which may arise during periods between sugar consumption (DiNicolantonio et al. 2018).

Prolonged excessive sugar intake can lead to increased tolerance within the dopamine system, indicating that greater quantities of sugar are required to achieve the same level of pleasure. This phenomenon is akin to that observed in drug addiction in which individuals become increasingly dependent on sugar for satisfaction and gradually lose interest in other rewards. The interplay between sugar addiction and the dopamine system is complex; however, this system plays an important role in this dynamic.

In summary, appetite regulation and neuromodulatory pathways are complex systems that involve the synergistic action of multiple signaling pathways and brain regions. Understanding the importance of these regulatory mechanisms provides more comprehensive insights into sugar addiction. This understanding can facilitate subsequent research on relatively safe and effective treatments for this condition, providing a theoretical basis for preventing and treating the psychological aspects of related disorders.

## 3 Manifestations of Sugar Addiction

The behavioral characteristics of sugar addiction include intense sugar cravings even in the absence of hunger, inability to control appetite, difficulty suppressing the desire for sugar, and inability to exercise self‐control, even when aware of the negative health impact. Excessive intake refers to the consistent consumption of large amounts of sugar, exceeding normal dietary requirements. This intake can lead to the development of psychological dependence on sugar and the belief that consuming sugar will relieve anxiety, stress, or negative emotions—similar to the effects of tobacco. This reliance may result in the suppression of other appetites, resulting in sugar overconsumption that diminishes the desire for other healthier foods, ultimately leading to an unbalanced diet. Physiological reactions such as fluctuations in blood sugar levels, decreased energy, or emotional fluctuations can occur following sugar consumption. Over time, individuals may seek more intense stimuli, requiring larger quantities or sweeter foods to achieve the same pleasurable sensation. These behavioral traits may indicate a tendency toward sugar addiction, which may negatively affect daily life, health, and mood.

Compulsivity and impulsivity are the two main elements in the complex cycle of addiction, which can manifest in three successive phases: binge/intoxication, withdrawal/negative emotions, and preconception/expectation, also known as the “craving phase.” Impulsive individuals may be less sensitive to the long‐term negative consequences of their behaviors, leading to a loss of control over food and drug intake and increased sensitivity to immediate gratification. This intolerance of delayed rewards is a prominent form of impulsivity and serves as a behavioral mechanism that highlights the relationship between impulsivity and addiction.

Uncontrolled drug use, drug cravings, and a high relapse rate—even after years of abstinence—are the hallmarks of substance addiction. Cue responsiveness and reward impulsivity are two essential characteristics of addiction that are crucial for the onset, maintenance, and recurrence of substance addiction. Understanding the neurochemistry underlying cue reactivity and reward impulsivity is crucial for gaining comprehensive insights into drug usage and relapse because these two factors have a significant impact on drug addiction. Notably, the dopamine and opioid systems are two neurotransmitter systems implicated in addiction (Weber et al. 2016).

## 4 Effects of Prolonged Intake of High‐Sugar Foods

### 4.1 Neurological Changes

Sugar preference often exceeds the normal range of appetite, leading to a phenomenon similar to drug addiction. Changes in the neurological system are also closely associated with this tendency. The effects of sugar addiction on neural systems are discussed in this section.

First, as mentioned above, consumption of high‐sugar foods affects the activity of the dopamine system in the brain. Consumption of high‐sugar food triggers the release of high dopamine levels in the brain, resulting in feelings of pleasure and satisfaction, sugar cravings, and dependence. Prolonged exposure to high‐sugar foods may lead to increased tolerance within the dopamine system, necessitating larger amounts of sugar to achieve the same pleasurable sensation, exacerbating the risk of sugar addiction.

Second, sugar addiction affects neuroendocrine systems associated with coping with stress, such as adrenaline and cortisol. Under stress conditions, hormones, such as adrenaline and cortisol, are released to mitigate stress. However, chronic intake of high‐sugar foods can disrupt the intricate balance of these hormones, thereby reducing the body's ability to cope with stress and increasing the risk of anxiety and depression. Bingeing on sucrose and food, followed by fasting, can induce anxiety and alter the balance of dopamine and acetylcholine in the nucleus ambiguus (Avena, Bocarsly, et al. 2008; Avena, Rada, et al. 2008). In addition, a high‐sugar diet can affect anxiety levels as well as post‐absorptive satiety and gut signaling related to food intake inhibition, leading to the downregulation of striatal D2 receptor expression (Onaolapo et al. 2020). Colantuoni et al. (2002) reported decreased D2 receptor binding in the striatum and increased D1 receptor binding in the NAc via radioautography demonstrated that rats intermittently exposed to sucrose exhibit decreased D2 receptor binding in the NAc. Moreover, rats that ingested large amounts of sugar showed a significant decrease in enkephalin mRNA levels in the NAc, as demonstrated by Spangler et al. (n.d.). This dopamine/acetylcholine neurochemical imbalance has also been observed in rats intermittently exposed to sugar when naloxone was administered to binge‐eating animals to induce opioid withdrawal (Colantuoni et al. 2002), as well as after 36 h of complete food deprivation (Avena, Rada, et al. 2008).

Furthermore, the cortical and amygdala regions of the brain are impacted by sugar addiction. These regions are closely linked to functions such as decision‐making, emotion regulation, and memory. Long‐term sugar overconsumption can cause aberrant activity in several brain areas, affecting the behavior and emotional stability of individuals. Notably, some studies have linked sugar addiction with anxiety, depression, and cognitive decline.

Overall, sugar addiction causes changes in several aspects of the nervous system, including increased activity of the dopamine system, disturbances in the balance of stress hormones, and abnormalities in cortex and amygdala functioning. These effects not only exacerbate sugar dependence but can also lead to various health problems and psychological distress. Therefore, in addition to fostering personal willpower, effective interventions for sugar addiction are needed. These should include dietary adjustments, changes in lifestyle habits, and professional assistance to restore the balance and health of the nervous system.

### 4.2 Changes in Brain Structure and Function

Sugar addiction can lead to changes in brain structure and function. One study suggested that high intake of high‐ingredient fructose may also be detrimental to the hippocampus, a brain region critical for memory and learning (Mazzoli et al. 2021). Neural changes in the hippocampus, accompanied by an overall decrease in the proliferation and differentiation of newly generated neurons in the dentate gyrus, are associated with hippocampus‐dependent learning and memory deficits. According to Beecher et al. (2021), long‐term high intake of sugar increases the likelihood of chronic ADHD and neurocognitive deficits in adulthood. They indicated that chronic (12 weeks) administration of higher sucrose concentrations (25%), but not lower concentrations (5%), in adolescent mice could enhance hyperkinetic responses to novelty and result in deficits in adult episodic and spatial memory. Another study confirmed the effect of high fructose on hippocampal inflammation has been confirmed by examining the inhibition of Ser 307 phosphorylation of hippocampal insulin receptor substrate 1, along with the levels of nuclear factor kappa B proteins and the mRNA levels of related inflammatory proteins (Stanhope et al. 2009). Moreover, Wong et al. (2017) demonstrated that 2 h of sucrose consumption over 24 days is sufficient to elicit spatial memory deficits, as measured via location recognition in rats with spatial deficits. They also reported that rats participating in a delayed discounting task exhibited behavioral evidence of hippocampal dysfunction. Witek et al. (2022) demonstrated that maternal consumption of a high‐sugar diet (sucrose or fructose) could be considered a potential new risk factor for neurobehavioral disorders, inducing behavioral phenotypes similar to ADHD in the offspring, characterized by increased motor activity and impulsivity. In addition, chronic consumption of high‐sugar meals may weaken anti‐inhibitory control, leading to diminished function of the prefrontal cortex, which is a part of the brain involved in self‐control and decision‐making. In turn, this may lead to greater difficulty in suppressing sugar cravings. Neuroadaptation occurs as the brain adjusts to the effects of high‐sugar diets over time, requiring greater sugar quantities to yield the same pleasurable feelings. This adaptation increases the likelihood of developing sugar addiction.

Overall, sugar addiction may alter neural circuits related to reward, control, and cognitive functioning, increasing the likelihood that individuals will overindulge in high‐sugar foods, which could lead to various detrimental repercussions.

### 4.3 Obesity

Sweetening has been linked to the increased consumption of nutrient‐dense foods and beverages and greater palatability. Consumption of diets rich in sugar or sugary drinks is linked to an increased risk of obesity, diabetes, and cardiometabolic disorders, despite reports suggesting that sugar may promote healthy eating. There are growing concerns regarding the potential long‐term effects of sugar‐ or sugar‐rich foods and beverages on brain reward systems, body composition, satiety, appetite, and food addiction (Onaolapo et al. 2020).

The hedonic, cognitive, and homeostatic feedback systems of an individual interact with each other to contribute to obesity, with the primary outcome of this interaction being excess energy that leads to increased body fat storage. Moreover, environmental and genetic factors play important roles in this complex interplay (Horton et al. 2023). Over the past four decades, the global prevalence of overweight and obesity has emerged as one of the most serious public health challenges in the 21st century. According to recent research, sugar and sugar‐related substances such as high‐fructose corn syrup and/or sugar‐sweetened beverages may significantly contribute to the development of diabetes, metabolic syndrome, and obesity. Moreover, the consumption of sugar‐rich meals and beverages increases the risk of obesity and related metabolic disorders such as insulin resistance, dyslipidemia, and hypertension.

Sugar can also induce a reprocessing effect in the brain, leading to increased food intake and preference. Increased sugar cravings are associated with excessive sugar intake and potentially addictive eating behaviors, which further aggravate body composition and lead to overweight and/or obesity. A tendency toward hypoactivity of the limbic dopamine system, particularly in the midbrain, characterized by decreased dopamine release and/or dopamine receptor density, may increase vulnerability to addictive disorders. According to this theory, relative dopamine deficiency can be compensated for by consuming addictive substances that increase its release. In this context, polymorphisms in the DA D2 receptor (*D2R*) gene have been demonstrated to correlate with reduced expression of this receptor, thereby increasing susceptibility to the development of substance dependence and obesity (Bumb et al. 2019).

Individuals with obesity often rate sweetness intensity greater than healthy controls. Functional magnetic resonance imaging studies have revealed that the brain reacts differently to carbohydrates, depending on their fructose content. Both epidemiological and interventional studies have indicated a link between sugar consumption and weight gain. Specifically, high‐sugar diets are more closely linked to weight gain than low‐sugar diets (Stanhope 2016). Moreover, obesity‐related alterations in reward‐related neural circuits are hypothesized to account for the altered sensitivity of individuals with obesity to food rewards, particularly owing to the reduced availability of striatal D2R. Sugar triggers brain reward circuits that stimulate the consumption of sugar‐rich foods through their appealing flavors and postprandial values.

### 4.4 Risk of Diabetes and Metabolic Disorders

Previous dietary intervention studies in humans have indicated elevated circulating lipid levels and reduced insulin sensitivity with the intake of high‐sugar diets compared to those observed in control diets (Stanhope 2016). However, both intracellular triglyceride levels and insulin metabolism are associated with diabetes (Bantle et al. 2000). Other studies have demonstrated that fructose intake can increase triglyceride concentration and decrease glucose tolerance and insulin sensitivity in healthy men. Hyperglycemia inhibits the effective adaptive immune response of macrophages and T cells, leading to various health issues. Diabetes‐related deaths are often associated with many complications, including obesity, cardiovascular disease, and diabetic retinopathy. Excessive glucose intake may compromise immune system function, thereby increasing the risk of morbidity and mortality in individuals with diabetes.

### 4.5 Cardiovascular Diseases

Hypertension and tachycardia were observed in rats fed an 8% sucrose solution instead of drinking water, and enhanced sympathetic activation was observed in the heart, pancreas, and hepatic organs of the sucrose‐fed rats. Moreover, chronic consumption of fructose may cause hypertension in rats and nocturnal hypertension in mice (Brown et al. 2008). High fructose levels can also cause hypertension in rats, with those fed a high‐fructose diet developing left ventricular hypertrophy, glomerular hypertension, and chronic kidney disease. These conditions are closely associated with high‐fructose diets, highlighting the potential cardiovascular and renal risks associated with high‐sugar diets.

### 4.6 Inflammation

Dietary sugars—mainly hexoses such as glucose, fructose, sucrose, and high‐fructose corn syrup—are commonly present in many diets. Excessive intake of these sugars can cause metabolic disorders and increase the levels of inflammatory mediators and pro‐inflammatory cytokines in various tissues, leading to insulin resistance and low‐grade chronic inflammation (Vasiljević et al. 2014). Low‐grade chronic inflammation may be induced by factors secreted by adipose tissue, inflammatory factors secreted by hepatic tissue, and increased intestinal permeability, all of which may ultimately lead to the development of cardiometabolic diseases. In high‐fructose‐fed rats, systemic markers of inflammation, including lipid transport protein‐2, e‐selectin, McP‐1, and PAI‐1, were upregulated, highlighting the inflammatory responses associated with excessive fructose intake (Cox et al. 2011).

Rheumatoid arthritis (RA), a common chronic systemic autoimmune disease caused by genetic, environmental, and endogenous factors, is characterized by systemic inflammation and persistent synovitis (Dey et al. 2020). Recent research has indicated that sugary drinks contribute to RA pathogenesis. Specifically, women who consumed $1 worth of sugary drinks daily had a higher risk of developing seropositive RA than those who did not consume sugary drinks, with women over the age of 55 being at greater risk (Hu et al. 2014). Sugar‐sweetened beverages may contribute to RA not only through autoimmune effects but also by altering the microbiome, which can affect downstream inflammatory pathways. Notably, high consumption of glucose, fructose, and sugar‐sweetened beverages reduces beneficial intestinal microbiota, particularly *Prevotella*, which is involved in the pathogenesis of RA.

## 5 Treatment of Sugar Addiction

### 5.1 Pharmacological Interventions

The beneficial effects of dopamine antagonists on reducing cue‐induced responses to sugar have been demonstrated (De Silva 2020). Cue responsiveness and reward impulsivity are two central aspects of addiction. As mentioned earlier, chronic consumption of high‐sugar foods can lead to compulsive and impulsive behaviors; therefore, targeting both these aspects using medication could potentially be beneficial for treating addiction. Amisulpride, a specific D2/D3 DA receptor antagonist, is particularly effective in individuals with heightened reward impulsivity and increased sensitivity to drug signaling. Notably, while naloxone exhibits modulatory effects, the two pharmacological agents are likely to exert distinct influences across emotional domains. This divergence suggests that further investigation is warranted to elucidate the interplay between affective states and reward‐related impulsivity under naloxone versus amisulpride administration. Therefore, the link between mood and reward impulsivity in the presence of these two drugs warrants further investigation (Weber et al. 2016).

In the endocannabinoid system, injections of cannabinoid type 1 receptor antagonists inhibit cue‐induced heroin and cocaine relapse. They can also reduce self‐administration of alcohol in animals with a history of alcohol dependence. However, there are insufficient data on the efficacy of cannabinoid type 1 receptor antagonists in individuals who consume large quantities of sugar‐sweetened beverages (Hajnal and Norgren 2001). Despite some evidence suggesting that supplements, including l‐glutamine and chromium picolinate, can reduce cravings for sweets, studies are limited. Currently, there is insufficient evidence to conclusively demonstrate the role of supplements in the management of sweet cravings.

Some natural herbs may effectively help control sugar‐dependence. For example, folic acid, a component of *Gymnema sylvestre* leaves, affects the acute consumption of high‐sugar sweeteners as well as hunger, pleasure, and cravings for more high‐sugar sweeteners. Moreover, *Sphecomyia vittata* significantly reduced the intake of high‐sugar sweeteners, decreasing the feelings of pleasure and satisfaction associated with consuming these foods. Notably, individuals with a sweet tooth enjoyed considerably less sweetness and lower cravings for high‐sugar sweetness after ingesting Muscadine leaves than those without a sweet tooth. However, it is important to assess the long‐term effects of Muskratula leaf‐derived products on overall sugar consumption and adherence to these interventions (Turner et al. 2020). Furthermore, ibogaine, which originates from the West African Tropical rainforest shrub *Tabernanthe iboga*, is an indole‐type alkaloid with neuroleptic effects. This evergreen shrub, along with other plants of the same genus, contains ibogaine, which has demonstrated a significant and lasting reduction in self‐administered drug use in animals. However, high doses of ibogaine are associated with side effects such as impaired motor function and cerebellar cell loss. Consequently, studies on its effect on sugar addiction are lacking (Belgers et al. 2016).

Finally, the most current non‐pharmacological approach involves transcranial stimulation of the prefrontal region of the brain by applying a direct current to the frontal region, resulting in impulsive reduction in a range of addictive behaviors. However, the optimal treatment frequency and duration remain unknown (Lee and Owyang 2017).

In recent years, progress has been made in the development of drugs to treat food addiction. However, there are limited treatments that specifically target sugar addiction. Future research should delve deeper into effective strategies to address sugar addiction, paving the way for more widespread and targeted interventions.

### 5.2 Psychological Interventions

Psychological interventions play a key role in reducing sugar addiction. Sugar addiction involves cravings and excessive sugar intake that can negatively affect physical and mental health. Therefore, psychological interventions and strategies can help patients cope with and reduce their addictions. For example, cognitive behavioral therapy can help people recognize and change harmful patterns of thinking and behavior that lead to sugar addiction, ultimately allowing them to develop healthier habits and responses. Psychoeducation involves providing information about the effects of sugar on the body, mechanisms of addiction, and importance of reducing sugar intake. This approach can help increase awareness and commitment among individuals. Emotional regulation focuses on teaching effective emotion regulation techniques to help individuals cope with their emotions in healthier ways, rather than seeking comfort or releasing stress through sugar consumption. Replacement strategies involve assisting individuals in finding alternative healthy activities or foods to replace excessive sugar consumption, such as exercising, drinking water infused with fruits, and incorporating more vegetables into their diet. Goal setting and rewards establish clear goals and create incentives that motivate individuals to stay on track with their efforts to reduce their sugar intake. A support system, including family, friends, or professional mental health practitioners, can provide essential encouragement and assistance in overcoming challenges. Moreover, a gradual reduction in sugar intake, rather than abrupt withdrawal, can reduce withdrawal symptoms and increase the likelihood of success. Self‐monitoring, which involves tracking eating habits and emotional states, helps individuals better recognize their sugar consumption patterns and make necessary adjustments and improvements.

These psychological interventions can be adapted and combined according to individual needs and circumstances to help effectively manage and reduce sugar intake. If sugar addiction is severe and affects quality of life, it is recommended to seek help and guidance from a professional mental health practitioner. Through health education, individuals can learn about the effects of sugar on health, including its effects on weight, diabetes risk, and cardiovascular disease. Education can also help individuals understand the importance of maintaining sugar intake within appropriate limits to maintain a healthy lifestyle. Therefore, preventive measures are crucial for targeting sugar addiction, including limiting the intake of high‐sugar‐containing foods and opting for healthier alternatives such as fruits, whole grains, and vegetables.

Developing a healthy eating plan and adhering to proper exercise are also important measures to prevent sugar addiction. Some individuals may seek sugary food to cope with emotional problems or stress. Psychological support and mental health services can help individuals cope with these challenges, find healthier ways to manage emotional stress, and reduce sugar dependence. Regular medical checkups are also important for detecting underlying health problems, such as diabetes or metabolic syndrome, which are associated with high‐sugar intake and sugar addiction. Notably, early detection and treatment can reduce health risks.

Overall, health education and preventive measures for sugar addiction are essential to promote healthy lifestyles and reduce the risk of chronic diseases. Through education and proactive measures, individuals can better manage their sugar intake and establish healthy eating habits to maintain physical and mental health.

### 5.3 Future Research Directions

There are various potential directions for future research to better understand sugar addiction. These include investigating neural mechanisms, genetic factors, behavioral interventions, food innovation, and mechanisms of sugar addiction. In‐depth investigations of the neural mechanisms underlying sugar addiction, especially how reward‐related areas of the brain are affected by sugar, are warranted to develop targeted interventions. Exploring the genetic basis of sugar addiction and the association between genes, sugar preferences, and the risk of addiction will help in the development of personalized prevention and treatment. Moreover, research on behavioral interventions for sugar addiction and exploring effective psychotherapy, cognitive behavioral therapy, and other strategies is essential to help individuals change their sugar consumption habits. Moreover, efforts should be made to promote the development of healthier, low‐sugar or no‐sugar alternatives; promote innovation in the food industry; reduce dependence on high‐sugar foods; enhance public awareness of the dangers of sugar addiction; advocate the concept of a healthy diet; and enhance awareness of self‐control over sugar consumption. Finally, elucidating the mechanisms and effects of sugar addiction will aid in preventing and treating sugar addiction and in promoting healthier eating habits and lifestyles.

## 6 Conclusion

High‐sugar intake stimulates the reward center in the brain, releasing dopamine, which produces a feeling of pleasure similar to the physiological mechanisms of drug addiction. Long‐term consumption of high‐sugar‐containing foods increases sugar cravings, withdrawal symptoms, mood swings, and psychological dependence on sugar. Sugar addiction leads to obesity, diabetes, cardiovascular disease, and other health problems that are harmful to the physical health of individuals.

## Author Contributions

**Jiayu Qi**: writing–review and editing; writing–original draft. **Fuqiang Shi**: supervision. **Zhihua Guo**: supervision. **Hongwu Li**: writing–original draft. **Di Qin**: supervision.

## Peer Review

The peer review history for this article is available at https://publons.com/publon/10.1002/brb3.70338.

## Data Availability

The data that support the findings of this study are available in PubMed at https://pubmed.ncbi.nlm.nih.gov/. These data were derived from the following resources available in the public domain: ‐ x‐mol, https://www.x‐mol.com/.
